# Frontiers in microfinance research for small and medium enterprises (SMEs) and microfinance institutions (MFIs): a bibliometric analysis

**DOI:** 10.1186/s43093-023-00195-3

**Published:** 2023-04-20

**Authors:** Francis Lwesya, Adam Beni Swebe Mwakalobo

**Affiliations:** 1 Department of Business Administration and Management, The University of Dodoma, Dodoma, Tanzania; 2 Department of Economics and Statistics, The University of Dodoma, Dodoma, Tanzania

**Keywords:** Microfinance, Microfinance institutions, SMEs, Microcredit, Bibliometric analysis

## Abstract

This article aims to present current research trends in microfinance for small and medium enterprises (SMEs) and microfinance institutions (MFIs), as microfinance plays an increasingly role in entrepreneurship development and poverty alleviation. The study uses a bibliometric analysis, in this work, we performed citation, bibliographic coupling, and keyword evolution analyses. The results show that research in microfinance for SMEs and microfinance institutions continue to grow. The authors found that recent research in microfinance for SMEs and microfinance institutions has evolved around eight thematic clusters, covering (1) access to and constraints on microcredit for SMEs (2) microfinance and economic empowerment, (3) sustainability of MFIs, (4) creditworthiness, microfinance technology infrastructure and financing patterns, (5) Islamic financial inclusion, (6) credit assessment models for microcredit, (7) microfinance and innovative business models, and (8) gender and equity crowdfunding. Research gaps in each of the thematic clusters are identified. Topics related to COVID-19, Islamic social finance, microfinance institutions, credit scoring models, crowdfunding, and entrepreneurial finance are likely to feature in the domain of microfinance and sustainability of MFIs in future.

## Introduction

Finance is widely acknowledged as one of the crucial resources for entrepreneurial development and poverty alleviation in developing countries. Resource-based view theory identifies three categories of important resources, namely (1) physical resources, (2) human resources, and (3) organizational resources [1]. These resources cover finance, organizational processes, people, and information (knowledge). This represents a synergy of resources that is important to the survival, growth, and development of any organization. However, small and medium enterprises (SMEs) continue to face significant obstacles to fulfilling their potential to grow, innovate, and create jobs due lack of finance and inadequate access to reliable sources of finance [2–4]. As a result, they fail to execute their strategies efficiently, grow, and build sustainable competitive advantages [5]. This failure is linked to several reasons, including the underdeveloped formal financial sector in many developing countries which is characterized by risk aversion, limited size, and a bias against small businesses, thus making the financial requirements of small businesses not sufficiently addressed by large financial institutions and banks [5, 6].

Over the past three decades, there has been widespread recognition of microfinance institutions (MFIs) and an increasing provision of microcredit services in developing countries in all economic sectors. These institutions provide a wide range of services, including loans, savings, insurance, and remittances, to the rural and urban poor through cooperatives, credit unions, specialty banks, commercial banks, and other institutional arrangements [7]. The previous goals of microfinance institutions have been to meet the financial needs of poor and marginalized members of society, such as women, through an increased outreach of services, and the sustainability of microfinance institutions [7]. MFIs are believed to be instrumental in poverty reduction initiatives by both governments and non-governmental organizations (NGOs) in developing countries, particularly in tackling social and financial exclusion. As a result, governments and public institutions instituted policies and strategies aimed at addressing the financial problems of the less privileged. This includes formulating microfinance policies, guidelines, and creating a conducive business environment for the creation of microfinance institutions (MFIs). The measures aimed to liberalize financial systems and attract more investment to the sector by lowering entry barriers. However, some of the MFIs still have rigid regulations, bureaucratic tendencies, charge high interest rates, lack sufficient capacity, governance, and transparency and accountability to act as responsible financial intermediaries [8–11]. These challenges raise doubts about the sustainability of microfinance institutions and microfinance services for the development of the SME sector. Therefore, in this article we consider the dynamic change in microfinance research for SMEs and microfinance institutions over the last six (6) years. Bika et al. [12] reported on the underdevelopment of research on entrepreneurial practices related to microfinance in developing countries. The existing and growing literature tends to focus on the relationship between entrepreneurial growth, microfinance, and institutional formalization [12–14]. Similarly, there exist several reviews and bibliometric works on the topic of microfinance in the literature, e.g., Kaushal et al. [15], Nisa et al. [16], and Ribeiro et al. [17]. However, most of these publications are topic and industry specific. For example, Kaushal et al. [15] discussed microfinance institutions and the empowerment of women. Nisa et al. [16] examined the effects of competition on microfinance institutions and Ribeiro et al. [17] examined whether microfinance promotes the development of its clients. The current research consolidates all studies on microfinance research for SMEs and microfinance institutions and suggests the likely future research direction. The specific goals were:

- To identify the most influential publications, authors, and institutions in microfinance research for SMEs and microfinance institutions
- What are the collaboration networks in microfinance research for SMEs and microfinance institutions?
- To understand the current research themes or topics in microfinance research for SMEs and microfinance institutions.

## Methodology

This study adopts bibliometric analysis using tools such as citation, bibliographic coupling, co-authorship, and keyword analysis to answer research questions and objectives [18]. Bibliographic coupling is used to identify current research trends and future priorities as they are reflected at the frontiers of research. It groups two documents with common references. In contrast to co-citation analysis, bibliographic coupling captures recent contributions, including future research direction [19]. On the other hand, co-authorship assesses the social ties between researchers, it captures the state of the research collaboration network within a field [20, 21]. Similarly, keyword analysis captures the most used words using keyword co-occurrence analysis [22].

### Data extraction process

We used Scopus for the collection of bibliographic data which is a large database covering over 20,000 peer-reviewed journals [23]. The search criteria and article selection are indicated in Table 1.

**Table 1:** Search criteria and article selection

		Reject	Accept
1	Filtering criteria		
	(a) Search engine: Scopus		
	(b) Search date: 25 August 2022		
	(c) Search term: ((“microfinance*” OR “micro finance” OR “micro-finance*” OR “microcredit*” OR “micro credit*” OR “micro-credit*”) AND (“microfinance institution*” OR “micro finance institution*” OR “micro-finance institution*” OR “mf*”) AND (“SMEs performance*” OR “SMEs success*” OR “small business performance*” OR “business performance*”))		52,342
	(d) Subject area and time frame: “Economics”, Business Management” and study time frame (2017–2022)	50,286	2056
	(e) Document type: Articles” and “reviews” (Excluded “Conference papers” “book chapters” and “books”)	276	1780
2	Article selection		
	(a) Erroneous records screening: include documents with valid authors information only	58	1722
	(b) Language screening: Include documents in English only	354	1368
3	Quality screening		
	(a) Content screening: Include articles if “Titles, abstracts and keywords” indicate relevance to scope of study (i.e., microfinance for SMEs and microfinance institutions only	1030	338

This table discloses a systematic procedure adopted to arrive at the final corpus of (388) articles for review

## Results and discussions

### Descriptive bibliometric analysis

Data collection shows that a final sample of 338 articles was written by 904 authors and were published in 205 journals. Most authors wrote multi-author documents (868 authors) and only 36 documents were single-authored (Table 2). This study covers a duration of 6 years.

**Table 2:** Main information about data collection

Description	Results
Timespan	2017:2022
Sources (Journals)	205
Articles (Documents)	338
Average years from publication	1.51
Average citations per documents	3.902
Average citations per year per doc	1.344
References	19,565
Authors	904
Authors of single-authored documents	36
Authors of multi-authored documents	868

**Fig. 1:**
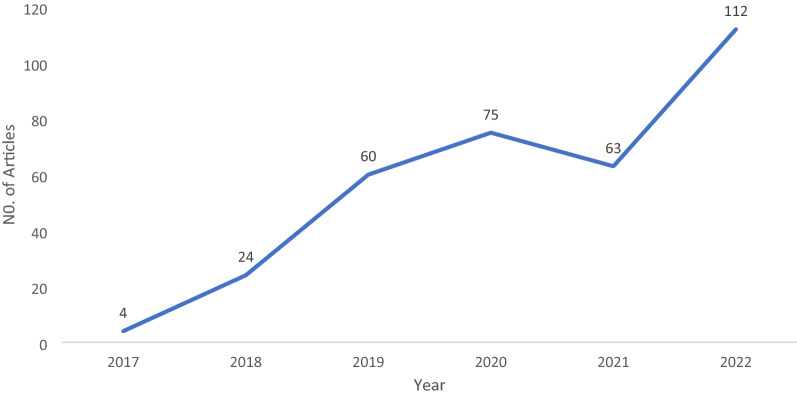
Number of articles per year

The level of item production in 2017 was slightly low with only four publications. However, the number of publications increased thereafter registering a total number of publications of 112 in August 2022 (Fig. 1). This suggests a growing research interests in microfinance research for SMEs and microfinance institutions.

The most important publications, authors, and institutions are listed in Table 3. Based on a set of citations, the most influential researchers in microfinance for SMEs and microfinance institutions are Chandio. A, Jiang Y. and Mohsin M. with 74, 72, and 51 citations, respectively. Based on several publications in microfinance for SMEs and microfinance institutions, Chandio. A is the most prolific researcher with three publications. In terms of institutions, the most influential institutions are Henan Agricultural University, Sichuan Agricultural University, and the Jiangsu University with 51, 51, and 51 citations, respectively. This shows that all top three institutions are based in China and recorded a similar number of citations and are majoring in the agriculture sector. This means that the agricultural sector is one of the most important sectors in need of microfinance support. In terms of intellectual contribution, the countries with the highest total number of publications and citations are the USA, China, and the UK with 54 (310), 29 (168), and 22 (161), respectively.

**Table 3:** Top 10 authors, institutions, and countries

TC	Authors	TP	TC	Organization	TP	TC	Country	TP
74	Chandio. A	3	51	Henan Agricultural University	1	310	USA	54
72	Jiang Y.	2	51	Sichuan Agricultural University	1	168	China	29
51	Mohsin M.	1	51	Jiangsu University	1	161	UK	22
51	Pathan A.	1	46	Universiti Utara Malaysia (UUM)	1	150	Australia	20
51	Rehman A.	1	39	University of the West of England	1	130	Malaysia	33
51	Twumasi M.	1	39	Carleton College, USA	1	117	India	46
46	Bin-Mohammad H.	1	39	Iza, Bonn, Germany	1	89	Germany	13
46	Shahar H.	1	36	Charles Stuart University	1	72	Netherlands	9
46	Ul-Hameed W.	1		University of Newcastle		57	Pakistan	14
39	Mersland R.	2	36	Western Sydney University	1	57	Belgium	4

*TC* total citation, *TP* total publications, the research constituents (i.e., author, institution, country)

The main journals published in microfinance and microfinance institutions are listed in Table 4. Based on citations, the most influential journals are Review of International Political Economy, Journal of Business Ethics, and Journal of Asian Business and Economic Studies with 64, 58, and 51 citations, respectively. The first two journals are rated A by the Australian Business Dean Council’s Journal Quality List 2020 (ABDC). Most articles are published in leading journals hosted by publishers such as Elsevier, Taylor and Francis, Emerald Insight, Springer Open, and Wiley Online Library.

**Table 4:** Most cited journals for topics in microfinance and microfinance institutions

No.	Source	TC	TP	ABDC	2017–2019	2020–August 2022
1	Review of International Political Economy	64	4	A	3	1
2	Journal of Business Ethics	58	5	A	1	4
3	Journal of Asian Business and Economic Studies	51	1	N/A		1
4	Management Science Letters	49	2	N/A	1	1
5	World Development	48	6	A	3	3
6	Applied Economics	41	11	A	4	7
7	Journal of Business Venturing	39	2	A*	1	1
8	Economic Modelling	37	3	A	2	1
9	Journal of Small Business Management	36	1	A		1
10	Mis quarterly	32	1	A*	1	
11	Journal of Small Business and Enterprise Development	30	4	C		4
12	Empirical Economics	28	4	A	1	3
13	International journal of Islamic and Middle Eastern Finance and Management	24	6	B	1	5
14	Emerging Markets Finance and Trade	24	2	B	1	1
15	Journal of Business Venturing Insights	24	1	A		

### Thematic clusters of microfinance and microfinance institutions research through bibliographic coupling

Using bibliographic coupling, we analyze the intellectual structure and recent knowledge development of the literature (Table 5). Bibliographic coupling captures the similarity between two documents based on the number of references they share [24, 25]. The bibliographic coupling analysis revealed seven clusters as follows.

**Table 5:** Thematic clusters of microfinance and microfinance institutions research

Theme	Author(s)	Title	TC
Cluster 1: Access to and constraints on microcredit for SMEs	Chandio et al. [26]	Determinants of demand for credit by smallholder farmers': a farm level analysis based on survey in Sindh, Pakistan	51
	Nguyen et al. [27]	SME credit constraints in Asia’s rising economic star: fresh empirical evidence from Vietnam	11
	Tran et al. [28]	Gender difference in access to local finance and firm performance: Evidence from a panel survey in Vietnam	10
	Silong and Gadanakis [29]	Credit sources, access, and factors influencing credit demand among rural livestock farmers in Nigeria	9
	Afari-Sefa et al. [30]	Determinants of smallholder vegetable farmers credit access and demand in southwest region, Cameroon	7
	Archer et al. [31]	SME credit constraints and access to informal credit markets in Vietnam	7
	Ademola Abimbola et al. [32]	Rotating and savings credit association (ROSCAS): A veritable tool for enhancing the performance of micro and small enterprises in Nigeria	6
	Diaz-Serrano and Sackey [33]	Microfinance and credit rationing: does the microfinance type matter?	6
	Ding and Abdulai [34]	Smallholder preferences and willingness-to-pay measures for microcredit: Evidence from Sichuan province in China	6
	Bairagya et al. [35]	Impact of Credit Accessibility on the Earnings of Self-employed Businesses in India	4
	Weng et al. [36]	Credit Constraints and Rural Households’ Entrepreneurial Performance in China	3
	Shuaibu and Nchake [37]	Impact of credit market conditions on agriculture productivity in Sub-Saharan Africa	3
Cluster 2: Microfinance and economic empowerment	Drori et al. [38]	How does the global microfinance industry determine its targeting strategy across cultures with differing gender values?	20
	Mia et al. [39]	History of microfinance in Bangladesh: A life cycle theory approach	14
	Bel hadj Miled et al. [40]	Can Microfinance Help to Reduce Poverty? A Review of Evidence for Developing Countries	8
	Samineni and Ramesh [41]	Measuring the Impact of Microfinance on Economic Enhancement of Women: Analysis with Special Reference to India	7
	Shoma [42]	Financing female entrepreneurs in cottage, micro, small, and medium enterprises: Evidence from the financial sector in Bangladesh 2010–2018	5
	Patel and Patel [43]	Does microfinance empower women from economic, social, and political perspectives? Empirical evidence from rural Gujarat	3
	Aslam et al. [44]	Impact of microfinance on poverty: Qualitative analysis for Grameen Bank borrowers	2
	Khan et al. [45]	Impact of Microfinance on Economic, Social, Political and Psychological Empowerment: Evidence from Women’s Self-help Groups in Kashmir Valley, India	2
	Nilakantan et al. [46]	On Ethical Violations in Microfinance Backed Small Businesses: Family and Household Welfare	2
	Okunlola et al. [47]	Empowering women through microfinance: Empirical evidence from Ibadan, Oyo state, Nigeria	2
	Patel and Patel [48]	Impact of microfinance on women empowerment: A study from the decision-making perspective	2
Cluster 3: Sustainability of Microfinance institutions	Gul et al. [49]	Performance of Microfinance Institutions: Does Government Ideology Matter?	20
	Awaworyi [50]	Microfinance financial sustainability and outreach: is there a trade-off?	18
	Cervelló-Royo, Guijarro et al. [51]	Social Performance considered within the global performance of Microfinance Institutions: a new approach	10
	Li et al. [52]	Convergence of the performance of microfinance institutions: A decomposition analysis	10
	Sinha and Pandey [53]	Efficiency of Microfinance Institutions in India: A Two-Stage DEA Approach	8
	Leite and Mendes [54]	To profit or not to profit? Assessing financial sustainability outcomes of microfinance institutions	8
	Abrar et al. [55]	Finance–growth nexus and banking efficiency: The impact of microfinance institutions	7
	Al-Azzam [56]	Financing microfinance institutions: subsidies or deposit mobilization	6
	Chikalipah [57]	Do micro-savings stimulate financial performance of microfinance institutions in Sub-Saharan Africa?	6
	Banto and Monsia [58]	Microfinance institutions, banking, growth, and transmission channel: A GMM panel data analysis from developing countries	4
	Adusei and Sarpong-Danquah [59]	Institutional quality and the capital structure of microfinance institutions: The moderating role of board gender diversity	3
	Tchuigoua et al. [60]	Lending and business cycle: Evidence from microfinance institutions	3
	Bharti and Malik [61]	Financial inclusion and the performance of microfinance institutions: does social performance affect the efficiency of microfinance institutions?	2
	Memon et al. [62]	Financial Sustainability of Microfinance Institutions and Macroeconomic Factors: A Case of South Asia	2
	Mia et al. [63]	Factors affecting borrowers’ turnover in microfinance institutions: A panel evidence	2
Cluster 4: Creditworthiness, microfinance technology infrastructure, and financing patterns	Bernards [64]	The poverty of fintech? Psychometrics, credit infrastructures, and the limits of financialization	34
	Tanima et al. [65]	Surfacing the political: Women’s empowerment, microfinance, critical dialogic accounting, and accountability	20
	Langevin [66]	Big data for (not so) small loans: technological infrastructures and the massification of fringe finance	13
	Masiak et al. [67]	How do micro firms differ in their financing patterns from larger SMEs?	11
	Wasiuzzaman et al. [68]	Creditworthiness and access to finance of SMEs in Malaysia: do linkages with large firms matter?	10
	Bongomin et al. [69]	Microfinance accessibility, social cohesion, and survival of women MSMEs in post-war communities in sub-Saharan Africa: Lessons from Northern Uganda	8
	Shahriar et al. [70]	Gender differences in the repayment of microcredit: The mediating role of trustworthiness	7
	Koh [71]	The impact of microfinance services on socioeconomic welfare of urban vulnerable households in Malaysia	2
	Mittal and Raman [72]	Financing woes: estimating the impact of MSME financing gap on financial structure practices of firm owners	2
Cluster 5: Islamic financial inclusion	Pomeroy et al. [73]	Financial inclusion to build economic resilience in small-scale fisheries	20
	Ali et al. [74]	Islamic financial inclusion determinants in Indonesia: an ANP approach	12
	Zauro et al. [75]	Enhancing socioeconomic justice and financial inclusion in Nigeria: The role of Zakat, Sadaqah, and Qardhul Hassan	8
	Raza et al. [76]	Determining the nexus between financial inclusion and economic development in Pakistan	8
	Baber [77]	Financial inclusion and FinTech: A comparative study of countries following Islamic finance and conventional finance	5
	Khmous and Besim [78]	Impact of Islamic banking share on financial inclusion: evidence from MENA	3
	Shaikh [79]	Using Fintech in scaling up Islamic microfinance	3
	Bolarinwa et al. [80]	Does Financial Development Really Matter for Poverty Reduction in Africa?	2
Cluster 6: Microcredit and credit assessment models	Shi et al. [81]	Exploring the mismatch between credit ratings and loss given default: A credit risk approach	18
	Liang and He [82]	Analyzing credit risk among Chinese P2P-lending businesses by integrating text-related soft information	13
	Enimu et al. [83]	Determinants of loan repayment among agricultural microcredit finance group members in Delta state, Nigeria	8
	Medina-Olivares et al. [84]	Spatial dependence in microfinance credit default	7
	Zhang et al [85]	Credit risk prediction of SMEs in supply chain finance by fusing demographic and behavioral data	6
	Mota et al. [86]	Determinants of microcredit repayment in Portugal: analysis of borrowers, loans and business projects	4
	Ala’raj et al. [87]	The applicability of credit scoring models in emerging economies: An evidence from Jordan	3
	de Paula et al. [88]	Estimating credit and profit scoring of a Brazilian credit union with logistic regression and machine-learning techniques	2
	Fuming et al. [89]	Micro- and small-sized enterprises’ willingness to borrow via internet financial services during coronavirus disease 2019	2
	Mou et al. [90]	Microlending on mobile social credit platforms: an exploratory study using Philippine loan contracts	2
	Wang et al. [91]	The role of social and psychological related soft information in credit analysis: Evidence from a Fintech Company	2
Cluster 7: Microfinance and innovative business models	Zhang et al. [92]	Exploring the Multi-Phase Driven Process for Disruptive Business Model Innovation of E-Business Microcredit: a Multiple Case Study from China	14
	Kimmitt and Dimov [93]	The recursive interplay of capabilities and constraints among microfinance entrepreneurs	9
	Souza et al. [94]	Multilevel latent class modeling to segment the microfinance market	4
	Kumra et al. [95]	Factors Affecting BoP Producer Intention to Use P2P Lending Platforms in India	2
	Singh and Dutt [96]	Microfinance and entrepreneurship at the base of the pyramid	2
Cluster 8: Gender and equity crowdfunding	Geiger and Oranburg [97]	Female entrepreneurs and equity crowdfunding in the US: Receiving less when asking for more	24
	Figueroa-Armijos and Berns [98]	Vulnerable Populations and Individual Social Responsibility in Prosocial Crowdfunding: Does the Framing Matter for Female and Rural Entrepreneurs?	7
	Zhao et al. [99]	Female entrepreneurs and equity crowdfunding: the consequential roles of lead investors and venture stages	3
	Cicchiello et al. [100]	In women, we trust! Exploring the sea change in investors’ perceptions in equity crowdfunding	2

*TC* total citation

#### Cluster 1: access to and constraints on microcredit for SMEs

This is the largest among all the eight clusters, it consists of 31 articles related with access to and constraints on microcredit for SMEs. The three most cited articles on this cluster are Chandio et al. [26], Nguyen et al. [27], and Tran et al. [28] with 51, 11, and 10 citations, respectively. The studies in this cluster show that factors such as formal education, company size, investments, financial assets, debt, equity, registration, sex, and age of the business owner significantly influenced the likelihood of credit constraints or demand [26, 27, 29]. Factors such as farming experience, size of land holdings, road access and advisory services, credit source information, deposits, household size, and marital status were important in the agribusiness sector [26, 29]. Likewise, gender was an important factor, as women were more restricted in accessing credit than their male counterparts, resulting in fewer women than men having access to formal credit [28, 29]. Other factors include lack of collateral, higher interest rates, rigid loan repayment schedules that limit access to microcredit and contribute to higher default rates [32, 33, 34, 37]. Initiatives such as empowering women in business leadership, strengthening credit agencies, membership of farmers’ unions, and agricultural extension services can increase both access to and demand for credit [28, 30]. Similarly, increasing the informal sector’s credit base by providing credit to specific segments of the informal credit market, as practiced in India, creating novel rural financial institutions, and establishing separate channels for lending to the most disadvantaged are proposed [31, 36, 35].

#### Cluster 2: microfinance and economic empowerment

This is the second largest cluster with 30 articles dedicated to microfinance and economic empowerment. Articles in this cluster discuss the role of microfinance in economic empowerment. In this cluster, studies show that areas with dominant microfinance access have experienced high levels of economic improvement [41, 44]. Similarly, access to microfinance by MFIs has benefited culturally excluded members of society, particularly women, thereby narrowing the gender gap in access to formal credit [38, 42, 43, 45]. However, high interest rates still prevent many women from obtaining credit [47]. Studies in this cluster also show that despite the positive impact of MFIs, the saturation of uncoordinated microfinance institutions and the expansion of multiple indebtedness have created challenges for regulators and management of microfinance institutions, leading to deterioration in loan portfolios and the financial sustainability of institutions [39, 41]. To enhance the sustainability of MFIs, effective MFI policies and improved regulatory regimes need to be put in place to enable MFIs to play a key role in poverty reduction [46, 47, 101].

#### Cluster 3: sustainability of microfinance institutions

This cluster consists of 27 articles related with sustainability of microfinance institutions. The three most cited articles in this cluster are Gul et al. [49], Awaworyi [50], and Cervelló-Royo et al. [51] with 20, 18 and 10 citations, respectively. The topics in this cluster deal with the financial sustainability of microfinance institutions (MFIs) for the thriving and growth of the microfinance industry. To achieve MFI sustainability, the complementarity between financial sustainability and outreach, the government’s positive ideology on MFI performance, social and technological innovation and financial deepening, and the exploration of digital technologies to increase operational efficiency are crucial factors [49, 50, 51, 52, 57]. We also observe different purposes between for-profit and not-for-profit microfinance institutions, while for-profit MFIs target relatively wealthier individuals and are therefore able to achieve wider outreach and charge higher interest rates than not-for-profit MFIs, the not-for-profit MFIs might have a smaller outreach and serve the poor more with lower interest rates [54]. Similarly, subsidies and deposit mobilization have been found to be a substitute fund with similar impacts on outreach and sustainability, lowering microcredit interest rates and allowing MFIs to reach poorer borrowers, but they both improve outreach and sustainability [56]. To improve sustainability, and institutional quality, most MFIs are targeting areas where they have a niche market or where commercial banks cannot serve low-income borrowers, and are trying to attract more start-ups and small industries, an activity which will potentially increase economic growth and the risk of insolvency of MFIs [55, 59].

#### Cluster 4: creditworthiness, microfinance technology infrastructure, and financing patterns

This cluster consists of 21 articles dealing with creditworthiness, microfinance technology infrastructure, and microfinance patterns. The three most cited articles in this cluster are Bernards [64], Tanima et al. [65], and Langevin [66] with 34, 20, and 13 citations, respectively. Studies in this cluster show that technological change and innovations in microfinance have increased efficiency in microfinance systems, this includes the use of big data technologies is trying to transform the fringe finance sector [66]. However, due to heterogeneity of MSMEs, the financing patterns of micro-enterprises differ significantly from that of larger SMEs. This explains to some extent why some MSMEs owners are reluctant to embrace mainstream funding [72]. It has also been found that alliances between SMEs and large companies do not have a major impact on overall creditworthiness, but do affect SME collateral and terms [67, 68]. Likewise, trustworthiness in microfinance is linked to gender differences, for example, female micro-borrowers have a better repayment record than male borrowers [70]. Similarly, it was found that in post-conflict communities in sub-Saharan Africa, social cohesion was used as a tool of social protection and as a safety net when female MSME borrowers lacked collateral and property rights [69].

#### Cluster 5: Islamic financial inclusion

This cluster consists of 18 articles related with financial inclusion. The three most cited articles in this cluster are Pomeroy et al. [73], Ali et al. [74], and Zauro et al. [75] with 20, 12, and 8 citations, respectively. Financial inclusion enhances the ability of people to engage in economic activities that lead to economic development and poverty reduction [76]. The themes in this cluster discuss about the barriers and determinants of financial inclusion. The identified barriers in this cluster relate to factors such as limited financial capability and literacy, lack of assets for collateral, geographic distance from a financial institution, and lack of formal identification [73]. Zauro et al. [75] proposed the use of Islamic financial instruments as means to enhance socioeconomic justice and financial inclusion in the Muslims’ communities. However, determinants of Islamic financial inclusion include financial literacy, religious commitment, socio-economy, and social influence, human capital, product and services, infrastructure readiness, and policies and regulation [74, 75]. Financial inclusion improves people’s ability to engage in economic activities that lead to economic development and poverty reduction [76, 80]. The themes in this cluster discuss the barriers and determinants of financial inclusion. Identified barriers in this cluster relate to factors such as limited financial capacity and literacy, lack of collateral assets, geographic distance from a financial institution, and lack of formal identification [73]. Zauro et al. [75] and Khmous and Besim [78] proposed the use of Islamic financial instruments as a means of enhancing socioeconomic equity and financial inclusion in Muslim communities. The determinants of Islamic financial inclusion include financial literacy, religious commitment, socioeconomics and social influence, human capital, products and services, infrastructure readiness, and policies and regulations [74, 75]. In terms of the performance of Islamic finance in Islamic countries compared to conventional finance, it has been equally successful with conventional finance. However, the percentage of women empowerment through financial inclusion in Islamic financial countries has surpassed the conventional financial sector in non-Islamic countries, thereby narrowing the gender gap. However, conventional finance is more advanced than Islamic finance in terms of the use of technology to provide financial services [77]. A study by Shaikh [79] proposes an integrative model embedding fintech on both the demand side and the supply side to enhance the reach, scale, and impact of Islamic microfinance services. This cluster suggests that more research needs to be explored in areas such as digital financial services in Islamic finance and financial sustainability issues.

#### Cluster 6: credit assessment models for microcredit

This cluster consists of 14 articles related with credit assessment models for microcredit. The three most cited articles in this cluster are Shi et al. [81], Liang and He [82] and Ali et al. [74], and Enimu et al. [83] with 18, 13, and 8 citations, respectively. The themes in this cluster discuss about credit assessment models for microcredit. A study by Liang and He [82] examines whether semantic textual information on the loan description helps in predicting the credit risk of different types of borrowers using a Chinese P2P platform. The results show that the semantic features of textual soft information significantly improve the predictability of credit scoring models and the promotional effect is most evident in first-time borrowers. One of the credit risk assessment tools is the loss given default (LGD) which is performed by minimizing the LGD for higher rated loans as a standard for risk rating in the sense that decreasing LGD is associated with higher creditworthiness from the creditors’ perspective of the borrower. This helps guide the way to solving the phenomenon of mismatch between credit ratings and LGDs in the existing credit rating literature [81]. de Paula et al. [88] also found that using statistical methods such as combining credit scoring and profit scoring makes it possible to provide credit to the customers with the highest potential for paying off credit union debt. Similarly, a study by Enimu et al. [83] argued that lenders should consider the socioeconomic determinants of group members to ensure sustainable loan repayment benefits. Factors such as age of group members, household size, household income and level of education, amount of credit received, length of stay in their community, distance to credit source, supervision, and disbursement are important in determining viability and sustainability of repayment [83]. Another method is the spatial random effects credit scoring model. It helps improve the ability to predict defaults and non-defaults for both individual and group loans, and several credit characteristics and demographic information are important determinants of individual loan defaults but not group loans [84]. On the other hand, to predict the credit risk of SMEs in supply chain finance (SCF), DeepRisk is proposed. This method applies the multimodal learning strategy to merge the two different data sources. The concatenated vectors derived from the data fusion are then used as input to the feed-forward neural network to predict SME credit risk. The fusion of the two different data sources is superior to existing approaches to SME credit risk forecasting in SCF [85]. Furthermore, three methods such as logistic regression (LR), artificial neural network (NN), and support vector machine (SVM) were compared to achieve the banks’ strategic and business goals. The results showed that the LR model outperformed both ANN and SVM on various performance indicators, including the achievement of the bank’s strategic and business objectives [87]. A study by Wang et al. [91] discusses the role of social and psychological soft information in predicting defaults in the P2P lending market and assesses the importance of such information in fintech lending analysis by combining hard and soft information on defaults. The results show that soft information can make a valuable contribution to credit assessment. Soft information shows high predictive power in our test, and in combination with hard information, it increases the power of our model to predict failures [91].

#### Cluster 7: microfinance and innovative business models

This cluster consists of eight articles related with microfinance and innovative business models. The three most cited articles in this cluster are Zhang et al. [92], Kimmitt and Dimov [93], and Souza et al. [94] with 14, 9, and 4 citations, respectively. The themes in this cluster discuss about microfinance and innovative business models for entrepreneurship development. A study by Kimmitt and Dimov [93], which uses Amartya Sen’s concepts of freedom of process and freedom of opportunity to understand microfinance and entrepreneurial behavior, found that microfinance institutions need to understand the needs of their customers in terms of a generative recursive mechanism that drives the chain of action and how entrepreneurs deal with their attitudes and intended relationships in practice. Souza et al. [94] discussed the importance of understanding each microfinance program and its clients on a case-by-case basis in order to use microfinance consumer market segmentation to develop the most appropriate strategies to address clients’ needs. Similarly, Kumra et al. [95] found that the faster access and ease associated with P2P lending positively influence borrowers’ intention to participate, lenders are positively influenced by the high returns and diversified risk. In addition, a study by Zhang et al. [92] proposes the use of new business models to leverage more opportunities to deliver customer value. This includes the use of e-business microcredit platforms. However, a thorough understanding of the models and their implementation is crucial to avoid disruptive business model innovations of e-business microcredit.

#### Cluster 8: gender and equity crowdfunding

This cluster consists of four articles dealing with gender and equity crowdfunding. The three most cited articles in this cluster are Geiger and Oranburg [97], Figueroa-Armijos and Berns [98], and Zhao et al. [99] with 24, 7, and 3 citations, respectively. The topics in this cluster discuss about relationship between gender and funding raised through equity crowdfunding. Geiger and Oranburg [97] using population data collected from US equity crowdfunding campaigns, found that campaigns receive significantly less funding when the main signatory is female. Regarding the interactions between gender and a campaign’s funding goal, their results show that campaigns raise significantly less funding as the target amount increases when the main signatory is female. Similarly, Figueroa-Armijos and Berns [98] found that applying for funds through a field partner that targets vulnerable populations can negatively impact the entrepreneur’s application for full funding. However, identifying the entrepreneur as female or rural as key characteristics of individual vulnerability increases the likelihood that the project will be fully funded. This study provides evidence that prosocial crowdfunding can indeed support the vulnerable and poor through a unique framing mechanism. Along the same lines, Zhao et al. [99] found that female entrepreneurs are more likely to be funded through equity crowdfunding than their male counterparts. The study found that lead investors placed the funding advantage for women entrepreneurs in the equity crowdfunding market. These results contribute to the literature on equity crowdfunding and female entrepreneurship by showing that an entrepreneur’s gender influences equity crowdfunding performance. This finding is supported by Cicchiello et al. [100] who found that having at least one woman on the board of companies seeking equity financing increases campaign success rates. The articles in this cluster suggest the existence of a relationship between gender and funding raised through equity crowdfunding.

Based on bibliographic coupling of thematic clusters, we present the research gaps and the future research direction (Table 6).

**Table 6:** Research gap and future directions based on clusters

Cluster	Gap	Research direction
Cluster 1: Access and constraints on microcredit for SMEs	Limited research on informal sources of credit and their impact on SMEs development	Explore the sustainability of informal sources of credit and their impact on SMEs performance and growth
	Inadequate research on measures or indicators of credit market conditions	Examine alternative measures of credit market conditions
	Limited research on the supply side constraints of credit	Study the link between credit constraints and business growth
		Research on the supply side constraints of credit for SMEs
		Examine the links between formal and informal sources of credit
Cluster 2: Microfinance and economic empowerment	Inadequate studies on regulatory and policy frameworks for access to microfinance for the poor	Research on the regulatory and policy frameworks for financial access to the poor
Cluster 3: Sustainability of Microfinance institutions	Limited studies on the association between MFIs outreach breadth and outreach depth in financial sustainability	Investigate whether there is a trade-off between outreach breadth and outreach depth when it comes to financial sustainability
	Insufficient research on the innovation and digital platforms implemented by MFIs	Explore the optimal level of outreach and financial sustainability where MFI is most efficient
		Explore digital platforms implemented by MFIs
Cluster 4: Creditworthiness, microfinance technology infrastructure and microfinance	Limited research on financing behavior and patterns of microfinance institutions	Research on funding models and patterns used by MFIs
Cluster 5: Islamic Financial inclusion	Inadequate research on Zakat, Sadaqah, and Qardhul Hassan model of Islamic financing	Explore the role of Zakat, Sadaqah, and Qardhul Hassan in closing the financial gap
	Insufficient research on comparative analysis between Islamic finance and conventional finance for financial inclusion	Conduct a comparative analysis between Islamic finance and conventional finance
Cluster 6: Credit assessment models for microcredit	Much research has been done on microcredit schemes in developing countries, where MFIs have adopted the group lending method for SMEs whose activities fall within the agricultural sector	Explore more research on MFI lending approaches and practices used for SMEs in other sectors such as manufacturing and service
	The studies focused on microcredit models based on past behavior, finance, and lifestyle, limited to corporate and bank lending	Explore different lending models including online lending platforms
		Research on various fraud detection methods to counter fictitious content of loan applications
Cluster 7: Microfinance and innovative business models	Limited research on the impact of peer-to-peer (P2P) lending in developing countries	Explore peer-to-peer (P2P) lending in developing countries
		Explore more business model innovation of e-business microcredit firms
Cluster 8: Female entrepreneurs and equity crowdfunding	Studies on crowdfunding are concentrated in developed countries	Explore entrepreneurship and crowdfunding in developing countries
		Less research on why campaigns with female primary signatories receives less funding
	The research has focused on equity crowdfunding	Explore other types of crowdfunding
		Explore the role of socioeconomic cultural context in equity crowdfunding

### Collaboration networks in microfinance research for SMEs and microfinance institutions

**Fig. 2:**
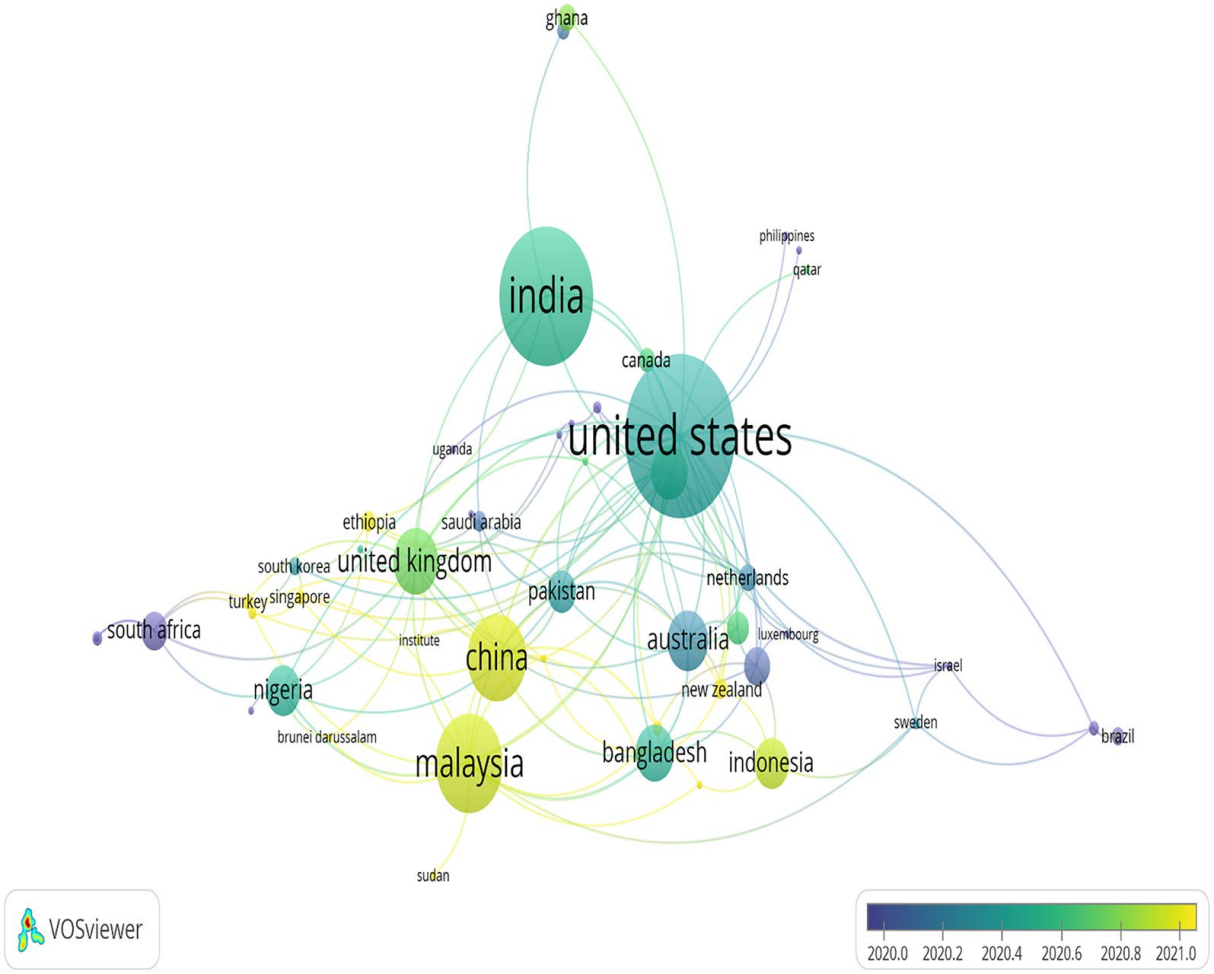
The author co-authorship network. The whole network consists of 51 nodes, 10 clusters, and 141 links. The total link strength value is 202

**Fig. 3:**
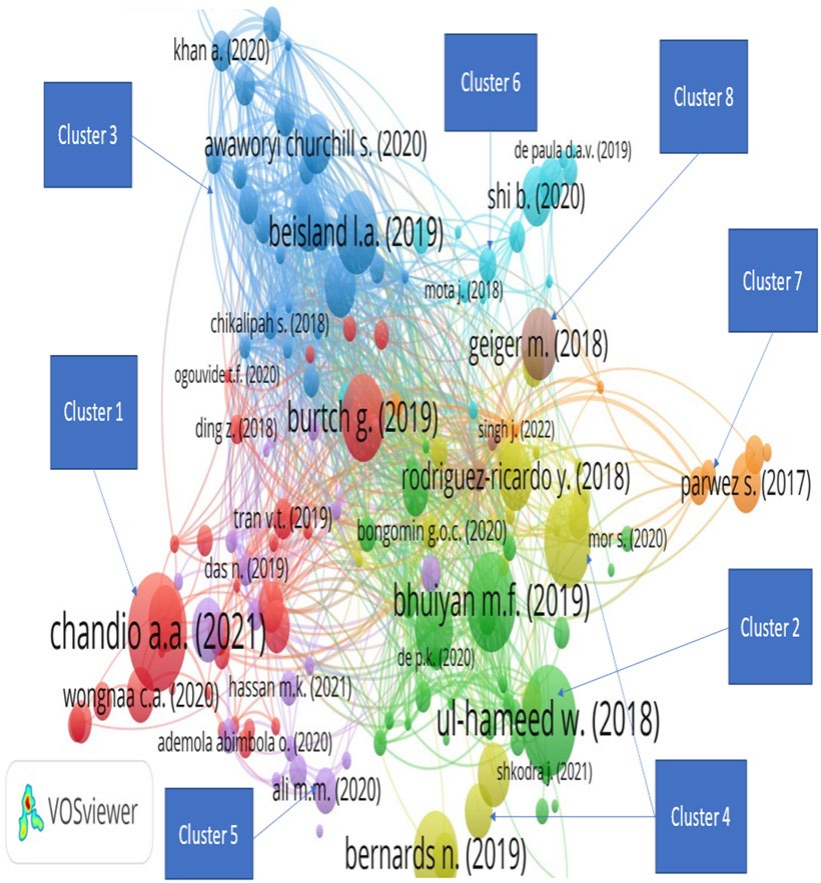
Bibliographic coupling network of publications

In terms of co-authorship and collaboration between authors and countries, the analysis shows that Chandio A., Jiang Y., and Kumar A. are the most influential authors in terms of overall link strength. Figure 2 shows the nodes representing author names, the links representing the co-authorship relationships between different authors, and the node sizes representing the publication counts of each author. Chandio. A is the most influential author with 74 citations. The data and network structure in Table 7 and Fig. 3 show that the research collaboration ties between developed economies and African countries is low. However, the analysis suggests that the collaborative network among developing countries is increasing. In terms of the country co-author network, Malaysia, Bangladesh, USA, and China are influential centers for research in microfinance for SMEs and microfinance institutions. Others are India, UK, and France. The cooperation relationship between the Malaysia and Bangladesh is the most common with six cooperations. USA and China follow with 6 cooperations.

**Table 7:** Top collaborating countries and authors

Collaboration network		TP	Author	TP	Citation	Total link strength
MALAYSIA	BANGLADESH	6	Chandio A.	3	74	6
USA	CHINA	6	Jiang Y.	2	72	6
AUSTRALIA	BANGLADESH	4	Kumar A.	2	15	5
AUSTRALIA	NEW ZEALAND	4	Mohsin M.	1	51	5
USA	GERMANY	4	Pathan A.	1	51	5
CHINA	PAKISTAN	3	Rehman A.	1	51	5
FRANCE	CANADA	3	Saroj S.	2	15	5
INDIA	USA	3	Sonkar V.	2	15	5
MALAYSIA	UK	3	Twumasi M.	1	51	5
USA	FRANCE	3	Block J.	1	11	4
AUSTRALIA	PAKISTAN	2	Chandran V.	1	14	4
CHINA	BANGLADESH	2	Kraemer-eis H.	1	11	4
CHINA	JAPAN	2	Lang F.	1	11	4
CHINA	NETHERLANDS	2	Lee h.-A.	1	14	4
CHINA	UK	2	Masiak C.	1	11	4
FRANCE	NETHERLANDS	2	Mia M.	4	17	4
GHANA	SPAIN	2	Moritz A.	1	11	4
INDIA	CANADA	2	Rahman m.	1	14	4
INDIA	FRANCE	2	Rasiah R.	1	14	4
MALAYSIA	NIGERIA	2	Abdullah A	1	10	3

### Thematic development of the microfinance research for SMEs and microfinance institutions using a conceptual thematic map

**Fig. 4:**
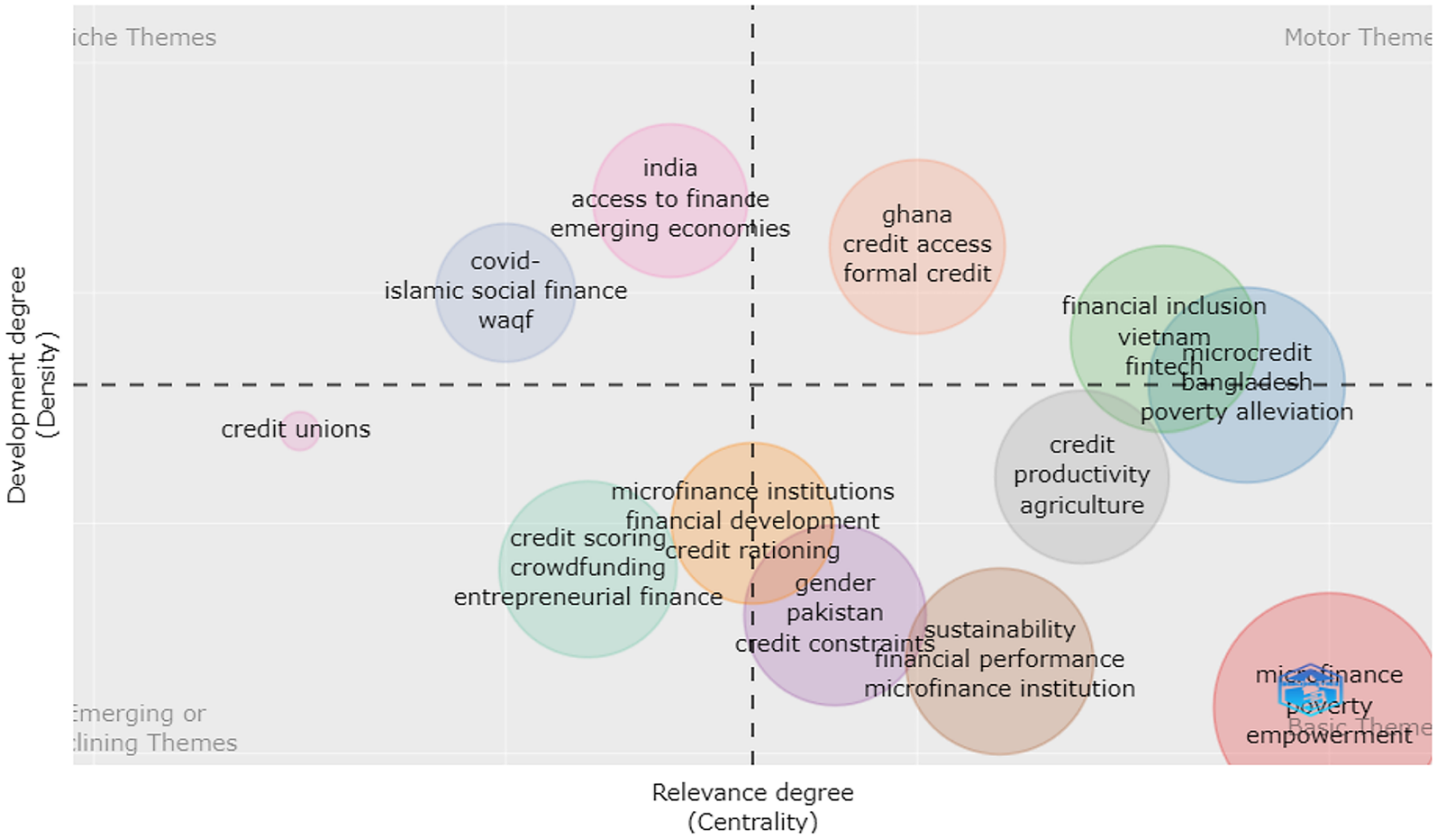
Conceptual thematic map

A conceptual thematic map was used to assess the thematic development of the microfinance research for SMEs and microfinance institutions based on keywords analysis (Fig. 4). The strategic diagram is divided into four quadrants (the upper right quadrant defines motor clusters, the upper left quadrant defines highly developed and isolated clusters, the lower left quadrant defines emerging or declining clusters, and the lower right quadrant defines fundamental and transversal clusters). Centrality measures the degree of a network’s interaction with other networks and can be understood as the external cohesion of the network, and density measures the internal strength of the network and can be understood as the internal cohesion of the network [102, 103].

#### First quadrant (motor themes)

This part presents well-developed themes that are of central importance for the structure of the research field. There are three bubbles in this quadrant, two of which traverse the fourth quadrant. Regarding to the Ghana corresponding bubble, the keywords with the highest frequency scores (occurrence) are; Ghana (6), credit access (5), and formal credit (4). Referring the bubble corresponding to financial inclusion, the keywords with the highest occurrence are; financial inclusion (24), Vietnam (8), and Fintech (6). Similarly, the bubble corresponding to microcredit, keywords with the highest frequency are: microcredit (38), Bangladesh (10), and poverty alleviation (10). The results of the two bubbles crossing the fourth quadrant suggest that they are well-developed themes that can structure the research field and are still the leading themes in broader microfinance research.

#### Niche themes (second quadrant)

These are well-developed and very specialized topics that are marginal in the overall field. This quadrant consists of two bubbles represented by India and COVID-19. In terms of the bubble corresponding to India, the top occurrence scores are: India (10), access to finance (4), and emerging economies (4). On the other hand, with the bubble corresponding to COVID-19, the highest occurrence scores come from COVID-19 (4), Islamic social finance (4), and waqf (3). The results suggest that issues in this quadrant (niche) such as access to finance, COVID-19, and Islamic social finance are potential topics that need to be more loosely linked to broader microfinance research. Researchers can explore these areas to advance knowledge in the broader field of microfinance.

#### Peripheral themes (third quadrant)

This quadrant consists of three bubbles represented by credit union, microfinance institution, and credit scoring. Credit union is the smallest bubble in this quadrant with only two occurrences. Regarding the bubble corresponding to credit scoring, the keywords with the highest frequency scores (occurrence) are: credit scoring (7), crowdfunding (6), and entrepreneurial finance (5). Similarly, with a bubble corresponding to a microfinance institution traversing the fourth quadrant, the highest frequency scores are: microfinance institution (13), financial development (5), and credit rationing (4). This bubble means that some of its components are fundamental and necessary for the development of the microfinance field. The results in this quadrant suggest that future research direction will continue to focus on topics such as microfinance institution, credit scoring, crowdfunding, credit union, entrepreneurial finance, and credit rationing.

#### Transversal and general basic themes (Four quadrant)

These are high-centrality, low-density themes that are important to the microfinance field but are not well developed. This includes four major bubbles represented by gender, sustainability, credit, and microfinance. Regarding the bubble corresponding to gender, the top occurrence scores are found for gender (15), Pakistan (7), and credit constraints (6). On the other hand, the bubble corresponding to sustainability, sustainability (11), financial performance (7), and microfinance institution (7) are the highest occurrence values. Similarly, the bubble corresponding to credit, credit (9), productivity (6), and agriculture (5) are the highest occurrence values. Lastly, the bubble corresponding to supply chain, supply chain (46), local food (11), and agriculture (8) are the highest occurrence values. On the other hand, the bubble corresponding to microfinance, microfinance (95), poverty (14), and empowerment (10) are the highest occurrence values.

## Conclusion, future research direction, and study limitations

This research is among the few studies covering microfinance research for small SMEs and microfinance institutions using bibliometric analysis. By applying bibliographic coupling, we found that recent research in this area has evolved around eight thematic clusters, covering (1) access to and constraints on microcredit for SMEs, (2) microfinance and economic empowerment, (3) sustainability of microfinance institutions, (4) creditworthiness, microfinance technology infrastructure and financing patterns, (5) Islamic financial inclusion, (6) credit assessment models for microcredit, (7) microfinance and innovative business models, and (8) gender and equity crowdfunding. The emerging research topics in the microfinance research for small SMEs and microfinance institutions relate to COVID-19, Islamic social finance, microfinance institution, credit scoring, crowdfunding, credit union, entrepreneurial finance, and credit rationing. Areas where research gaps remain include the sustainability of informal sources of credit and their impact on SME performance, financing models and patterns used by MFIs, the sustainability of Islamic finance, crowdfunding in developing countries, and regulatory and policy frameworks for MFIs. We have also observed that most microfinance research is focused on the agricultural sector and Western countries and Asian countries like China, Bangladesh, Australia, USA, and UK are dominating microfinance research lately and there is less research collaboration between Africa and Western countries. The limitations of the research are that is based on the bibliometric analysis and only one database, namely Scopus was involved. Secondly, the research conducted a retrospective review from 2017 to August 2022 to identify the current research trends, a wider inclusion of other databases and deeper content analysis could expand the findings of this research.

## Theoretical and managerial implications

The results of this study may be of practical interest to managers, industry researchers, and policy makers. For example, managers can apply various models and techniques to enhance sustainability of microfinance institutions such as increasing outreach, social and technological innovation, financial deepening, and the use of digital technologies to increase operational efficiency. Managers can also employ various efficient credit assessment models such as the loss given default (LGD). Similarly, managers can combine credit scoring and profit scoring makes it possible to provide credit to the customers with the highest potential for paying off credit union debt. Another method is the spatial random effects credit scoring model which helps to improve the ability to predict defaults and non-defaults for both individual and group loans. Industry researchers can use our research to understand the broad spectrum of research in the field, emerging research areas, research gaps, and future research direction. Similarly, policy makers can apply the outcomes to design policies and interventions in regions of the regulatory and policy frameworks for financial access to the poor and the sustainability of MFIs.

## Data Availability

Available.

## Abbreviations

MFIs: Microfinance institutions
LGD: Loss given default
SMEs: Small and medium enterprises
SCF: Supply chain finance
NN: Artificial neural network
ABDC: Australian Business Dean Council’s Journal Quality List
SVM: Support vector machine
LR: Logistic regression
